# A Rare Case of Thymic Mucosa-Associated Lymphoid Tissue (MALT) Lymphoma

**DOI:** 10.7759/cureus.91068

**Published:** 2025-08-26

**Authors:** Sheikh Izzat Bin Zainal-Abidin Bahajjaj, Cynthia Ming Li Chia

**Affiliations:** 1 Cardiothoracic Surgery, Singapore General Hospital, Singapore, SGP

**Keywords:** extranodal lymphomas, mucosa-associated lymphoid tissue (malt) lymphoma, robot-assisted, thoracic malignancy, thymic tumors

## Abstract

Thymic mucosa-associated lymphoid tissue (MALT) lymphoma is a type of diffuse B-cell lymphoma located in the thymus that is extremely rare. This case report illustrates the treatment of a 39-year-old Asian female patient with thymic MALT lymphoma. She presented with constitutional symptoms and chest pain. A CT scan of the chest revealed a soft tissue density in the right anterior mediastinum measuring 4×4.2 cm, likely originating from the thymus. After counseling between the options of surveillance imaging, CT-guided biopsy, or upfront surgery, the patient opted for surgical resection of the mass for both diagnostic and therapeutic intent. She then underwent robot-assisted excision of the mediastinal mass and total thymectomy on July 6, 2023. Intraoperative findings noted a 3 cm thymic mass without direct invasion into other local structures. The histological report revealed extranodal marginal zone lymphoma of MALT type. The patient was then referred to medical oncology, where it was determined that surgical resection of the tumor in her case was sufficient for oncological treatment, and no further radiotherapy or chemotherapy was required. The patient was also screened for possible autoimmune conditions and was found to be ANA positive, as thymic MALT lymphoma has known autoimmune associations with Sjögren’s disease and SLE, among others.

## Introduction

Thymic mucosa-associated lymphoid tissue (MALT) lymphoma is a rare subtype of B-cell lymphoma. It is a low-grade extranodal lymphoma most often found in the gastric tract [1]. However, thymic MALT lymphoma is particularly rare, with only about 5% of cases occurring in the thymus [2]. The thymus itself is a primary lymphoid organ and plays an important role in immune system development during childhood [3]. It develops from three germ cell lines and therefore has the potential to transform across multiple cell lines [4]. The earliest case report of thymic lymphoma was published in 1990 [5], and since then, only about 50 case reports, 13 case series, and one systematic review have been published on the topic [6].

## Case presentation

This is a case report of Mdm SH, a 39-year-old woman with a past medical history of herpes simplex virus meningitis, ascending colon diverticulitis, asthma, and prior tubal ectopic pregnancy.

She initially presented with left-sided chest pain associated with unintentional weight loss of approximately 5 kg (baseline weight 60 kg) and loss of appetite over three to four months. She had no fever, chills, rigors, or night sweats. Regarding family history, her younger sister was diagnosed with lymphoma, and her elder sister was diagnosed with breast cancer in her 30s. Physical examination was normal, with no palpable lymphadenopathy or organomegaly.

**Figure 1:**
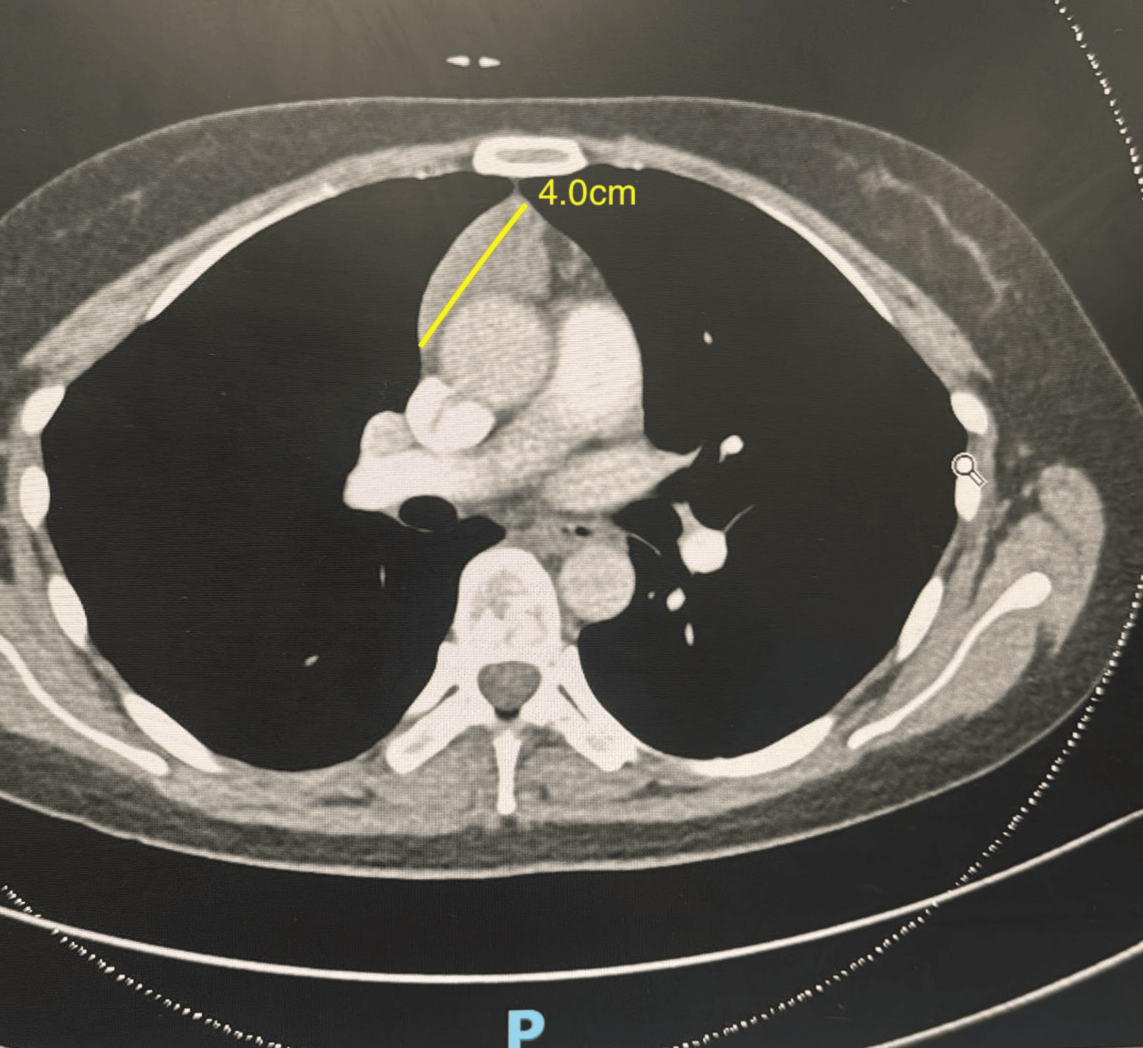
Initial CT image of the anterior mediastinal mass showing a triangular, homogeneous, hyperdense lesion without enhancing necrotic areas or lobulation.

Further investigation with contrast-enhanced computed tomography (CT) of the chest revealed an elongated triangular lobulated soft tissue density in the right anterior mediastinum measuring 4.0×4.2 cm (Figure 1). The lesion was not cystic or necrotic, although thymic in origin, and was not typical of a thymoma. Initial CT images also showed possible small areas of airspace opacification or airway thickening in the right middle lobe, likely representing airway-based inflammation or infection.

Laboratory investigations were performed (Table 1). Her full blood count was normal; however, she was noted to have elevated beta-2 microglobulin at 3896 µg/L. Notably, other tumor markers such as lactate dehydrogenase, alpha-fetoprotein, and beta-hCG were not elevated.

**Table 1:** Laboratory test results. Full blood count was normal; notably, beta-2 microglobulin and ANA were elevated.

Laboratory Tests			
Full blood count		Units	Range
Hemoglobin	13.7	g/dL	12.0-16.0
White blood cell count	6.72	x10^9^/L	4.0-10.0
Platelet count	224	x10^9^/L	140-440
Red blood cell count	4.66	x10^12^/L	4.2-5.4
Hematocrit	42.8	%	36-46
Mean corpuscular volume	88.4	FL	78-98
Mean corpuscular hemoglobin	29.4	PG	27-32
Mean corpuscular hematocrit	33.3	g/dL	32-36
Neutrophil	57.5	%	40-75
Lymphocyte	26.6	%	15-41
Monocyte	11.3	%	2-10
Eosinophil	3.6	%	0-6
Basophil	1	%	0-1
Neutrophils absolute	3.86	x10^9^/L	2.0-7.5
Lymphocyte absolute	1.79	x10^9^/L	1.0-3.0
Monocyte absolute	0.76	x10^9^/L	0.2-0.8
Eosinophils absolute	0.24	x10^9^/L	0.04-0.44
Basophil Absolute	0.07	x10^9^/L	0-0.10
Tumor markers			
Beta-2 microglobulin	3896	ug/L	878-2000
Alpha-fetoprotein	<2.7	ug/L	<7.1
LDH	256	U/L	222-454
Beta-HCG	<0.3	U/L	<5.3
Autoimmune marker			
ANA titer	>640		>640 (positive); >320 (weak positive); >160 (borderline)
ANA pattern	Speckled		

**Figure 2:**
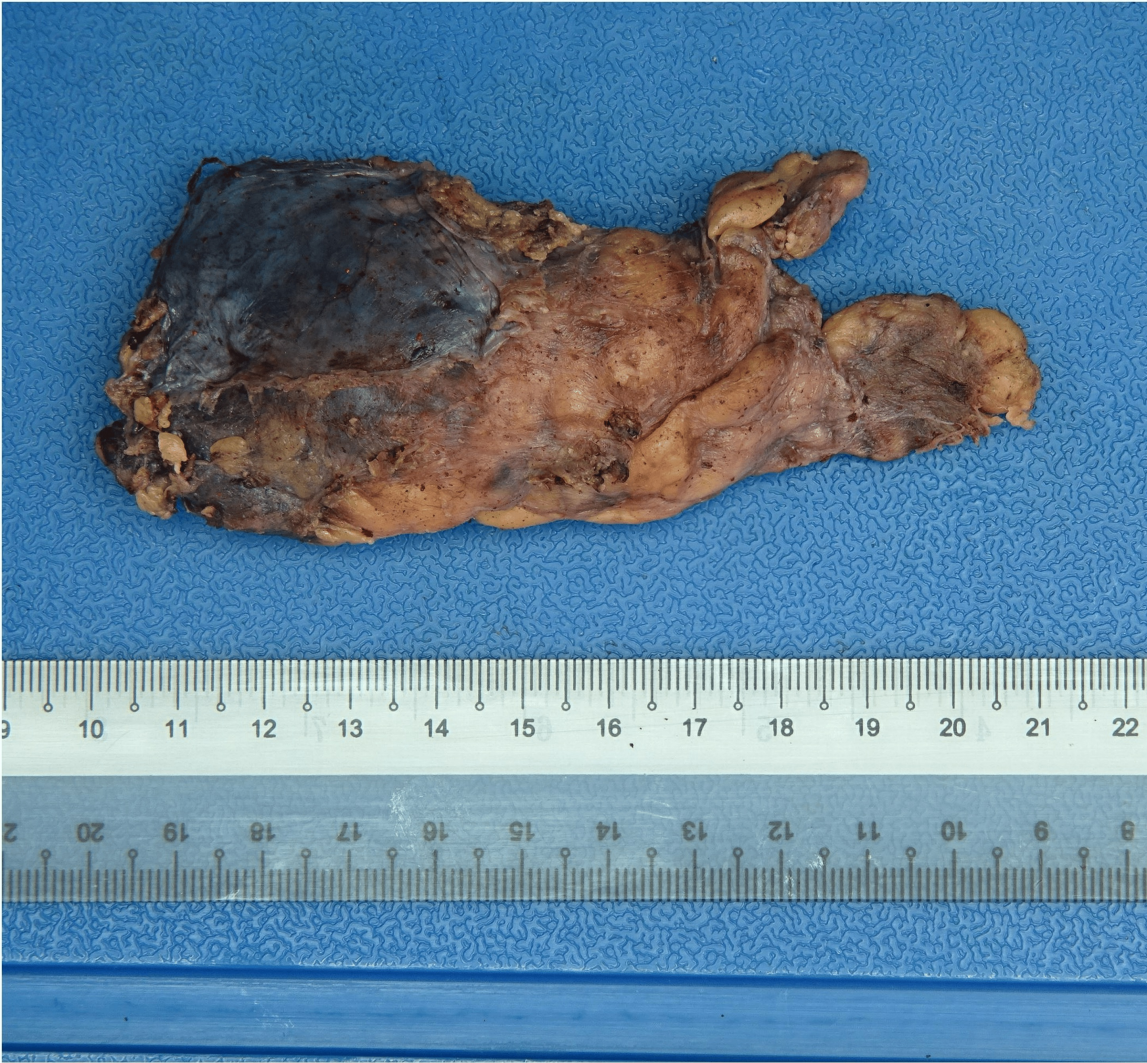
Gross tumor specimen.

On review in clinic, the patient was presented with the options of interval surveillance, CT-guided biopsy, or upfront surgery. She was counseled for surgery to remove the anterior mediastinal mass for both diagnostic and therapeutic intent. The patient then underwent robot-assisted excision of the anterior mediastinal mass and total thymectomy on July 6, 2023. The surgery was performed via a three-port robotic incision with one assistant port. Intraoperative findings noted a mass approximately 3 cm in size, with the total gross specimen resected measuring approximately 10 cm in length (Figure 2). There was no involvement or invasion of surrounding structures, and no lesions were seen in the lungs.

**Figure 3:**
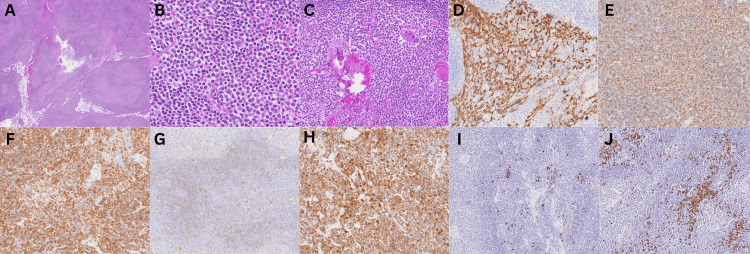
Histology and immunological stains of the resected specimen. A) H&E, 1x. Thymic tissue showing effacement of the architecture by an atypical lymphocytic proliferation B) H&E, 40x. Atypical lymphocytes comprising monocytoid and plasmacytoid cells C) H&E, 20x. Infiltration of thymic epithelium by atypical lymphocytes D) CK19, 20x. Presence of lymphoepithelial islands E) CD20, 20x. Highlighting atypical B lymphocytes F) BCL-2, 20x. Atypical lymphocytes showing diffuse positivity G) IgD, 10x. Disrupted IgD-positive mantle cells infiltrated by atypical lymphocytes H) P27, 20x. Diffuse positivity in the atypical lymphocytes I & J) Kappa (I), Lambda (J). Showing immunoglobulin light chain predominance

Histopathology showed extranodal marginal zone lymphoma of MALT type. Microscopically, the specimen comprised thymic tissue with extensive effacement by atypical lymphocytic proliferation (Figure 3A). The atypical lymphocytic proliferation consisted of nests of monocytoid and plasmacytoid cells (Figure 3B). The atypical lymphoproliferation demonstrated immunoreactivity with CD20, indicating a B-lymphocyte-predominant proliferation, and was diffusely positive for BCL2 (Figure 3E and Figure 3F). This was accompanied by diffuse cyclin-dependent kinase p27 nuclear positivity (Figure 3H). The proliferation index showed Ki-67 of 10%, and the IgG4/IgG ratio was <5%. These features favor marginal zone lymphoma.

CT of the thorax, abdomen, and pelvis noted no other distant lesions, and the patient was deemed to have Ann Arbor Stage 1 thymic MALT lymphoma. The patient was then referred to medical oncology, where she was offered further testing via bone marrow aspiration in view of her significant family history, which she declined. At a multidisciplinary tumor board discussion, as the mass was fully resected and no residual disease was noted on repeat CT imaging, it was determined that no further treatment was required, and the patient was scheduled for regular follow-up and surveillance. As thymic MALT lymphoma has known autoimmune associations, the patient was noted to be ANA positive (Table 1), suggesting a possible underlying undiagnosed autoimmune condition; however, she declined further assessment or evaluation.

## Discussion

Thymic MALT lymphoma is an indolent diffuse B-cell lymphoma. On imaging, particularly CT, it is difficult to distinguish from other thymic masses [7]. This partly explains why most diagnoses are made postoperatively, only after histology is obtained.

With regard to differential diagnosis, thymic masses are difficult to differentiate on imaging [8]. An abnormal appearance of the thymus may be caused either by diffuse enlargement of the gland or by a discrete mass, and there is considerable overlap in the features of thymic lesions on imaging [9]. Therefore, in cases such as this, imaging alone is insufficient for a firm diagnosis. Differentials would have included thymic hyperplasia, thymomas, thymic cysts, and lymphomas, among others [8]. The raised beta-2 microglobulin levels, along with the significant family history of lymphoma, increased the pre-diagnostic probability of lymphoma.

In this case, the patient underwent upfront surgery instead of a CT-guided biopsy for several reasons. Although biopsy is less invasive than surgery, if a CT-guided biopsy had demonstrated a malignant lesion, treatment options would still have included surgery or other modalities such as radiotherapy. Hence, upfront surgery may prevent the patient from undergoing multiple procedures, while also providing a safe and accurate method for tissue diagnosis [10]. Furthermore, with CT-guided biopsy, there is a risk of obtaining a non-diagnostic or non-representative sample, which is avoided with upfront surgery. Thymic MALT lymphoma appears to be a unique disease that usually develops only in the thymus, and thus complete surgical removal remains the mainstay of treatment [11]. From the patient’s perspective, given her significant family history, upfront removal of the mass was more acceptable despite the possibility that surgery could represent overtreatment if the lesion were benign. Therefore while CT-guided biopsy is an alternative for young patients with low surgical risk, atypical CT findings, and strong family history, upfront surgery should be considered.

Although thymic MALT lymphoma is largely a localized disease, in the rare cases with lymph node or systemic involvement, postoperative chemotherapy may be considered [12]. Given that the patient had Stage 1 disease, chemotherapy was not indicated. Regarding prognosis and long-term outcomes, these patients generally have an excellent prognosis, with a five-year overall survival rate of 97% [6]. The condition has recognized associations with autoimmune disorders, such as Sjögren’s syndrome and systemic lupus erythematosus, which are commonly found in patients with thymic MALT lymphoma [13]. This patient was noted to have laboratory features suggestive of an autoimmune condition, but declined further evaluation.

## Conclusions

In conclusion, we present a rare patient with a possible autoimmune disease, diagnosed with thymic MALT lymphoma after surgical resection of an anterior mediastinal mass arising from the thymus. The diagnosis of thymic masses on CT imaging is difficult, and hence biopsies are essential in the workup of such patients. In young patients with low surgical risk and significant family history, upfront total thymectomy is a safe way to achieve both diagnostic and therapeutic goals and can be considered as first-line management. Patients like these generally have a good prognosis and may not require further treatment post-surgery.
